# Assessment of venous congestion with venous excess ultrasound score in the prognosis of acute heart failure in the emergency department: a prospective study

**DOI:** 10.1093/ehjopen/oeae050

**Published:** 2024-07-10

**Authors:** Irene Landi, Ludovica Guerritore, Andrea Iannaccone, Andrea Ricotti, Philippe Rola, Marco Garrone

**Affiliations:** Dipartimento di Medicina Traslazionale, Università del Piemonte Orientale, Novara, Italia; Medicina Chirurgia d'accettazione e d'urgenza, Azienda Ospedaliera Ordine Mauriziano, Via Magellano 1, 10128 Torino, Italia; Medicina Interna e Unità di Terapia Semi Intensiva internistica, Azienda Ospedaliera Ordine Mauriziano, Via Magellano 1, 10128 Torino, Italia; Clinical Trial Unit, Azienda Ospedaliera Ordine Mauriziano, Via Magellano 1, 10128 Torino, Italia; Division of Intensive Care, Santa Cabrini Hospital, Montreal, QC, Canada; Medicina Chirurgia d'accettazione e d'urgenza, Azienda Ospedaliera Ordine Mauriziano, Via Magellano 1, 10128 Torino, Italia

**Keywords:** Congestive heart failure, Point-of-care ultrasound, VExUS score, Venous congestion

## Abstract

**Aims:**

In acute decompensated heart failure (HF), systemic venous congestion contributes to patients’ symptoms and hospital admissions. The purpose of our study is to determine if venous congestion, examined using the venous excess ultrasound (VExUS) score, predicts HF-related hospitalization and mortality in patients admitted to the emergency department (ED) with acute decompensated HF.

**Methods and results:**

Fifty patients admitted for acute HF in ED underwent ultrasound (US) assessment according to the VExUS score within the first 24 and 72 h. All patients were followed up with a telephone call at 30 and 60 days after hospital discharge. On admission, 56% had a VExUS score of 3. After 72 h, 32% had no more signs of congestion at the Doppler VExUS examination (inferior vena cava < 2 cm, VExUS score of 0); a similar percentage still exhibited a VExUS score of 3 despite therapy. Eighty per cent of patients were hospitalized after admission to the ED, while six (15%) died in-hospital; all exhibited a first-assessment VExUS score of 3. No patient with a VExUS score < 3 died during the study. During short-term follow-up, 18 patients were readmitted to the ED for acute decompensated HF. Ninety-four per cent of the readmitted patients had a VExUS score of 3 at the Doppler assessment at the first ED admission.

**Conclusion:**

Severe venous congestion, defined as a VExUS score of 3 at the initial assessment of patients with acute decompensated HF, predicts inpatient mortality, HF-related death, and early readmission.

## Graphical Abstract

**Graphical Abstract:**
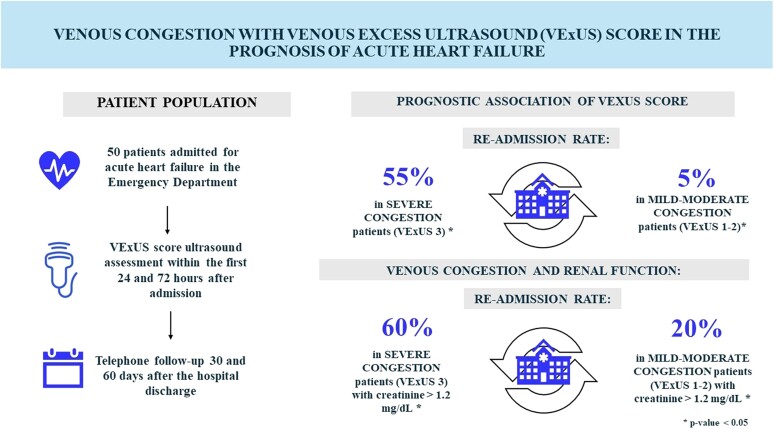

## Introduction

It is well known that, in patients with acute decompensated heart failure (AHF), systemic venous congestion contributes to patients’ symptoms and hospital admissions, while growing evidence suggests that congestion drives disease progression.^1,2^ In addition, systemic venous congestion harms organs such as kidneys and liver due to dysfunctional organ perfusion. Diuretics and vasodilators remain the mainstay of treatment, while ultrafiltration has emerged as an invasive option for patients with diuretic resistance.^3^ Early detection may allow immediate intervention before full-blown congestion, and thus organ damage, is established. A multimodal bedside ultrasound (US) strategy for assessing haemodynamic and filling status should include echocardiography, lung US, and systemic vein US.^4,5^ This integration is critical in diagnosing, monitoring, and analysing the response to therapeutic measures of venous congestion. The venous excess ultrasound (VExUS) score, as devised by Beaubien-Souligny *et al.*, has been designed to evaluate the performance of several venous congestion grading systems based on multi-vessel Doppler patterns to predict acute kidney injury (AKI) in patients undergoing cardiac surgery. They found that severe congestion, defined as severe Doppler anomalies in at least two of the hepatic, portal, and renal veins, was the best predictor of AKI defined by the Kidney Disease Improving Global Outcomes criteria.^6^

The purpose of our study is to determine if venous congestion, examined using the VExUS protocol, predicts HF-related hospitalization and mortality in patients admitted to the emergency department (ED) and diagnosed with acute decompensated HF. As a secondary endpoint, we assess whether biochemical markers of organ dysfunction [creatinine, estimated glomerular filtration rate (eGFR), and brain natriuretic peptide (BNP)] are associated with venous congestion and, consequently, with worse outcomes [readmission to the ED or death for a new episode of acute HF].

## Methods

This was a prospective, monocentric, study performed in the ED of the secondary care community Hospital ‘Mauriziano’ in Piedmont, Turin, Italy. The study was conducted in accordance with the Declaration of Helsinki and approved by the Hospital Research Ethics Committee. An informed consent was obtained for each patient. We enrolled 50 patients admitted with a diagnosis of AHF according to the European Society of Cardiology guidelines^3^ in the ED presenting with acute respiratory insufficiency (partial pressure of oxygen < 60 mmHg or oxygen saturation ≤ 92%) and BNP ≥ 500 pg/mL. Exclusion criteria were age < 18 years, waived study consent, inadequate acoustic windows, haemodynamic instability, cirrhosis with portal hypertension, inferior vena cava (IVC) thrombosis, Stage V chronic kidney disease (eGFR < 15 mL/min/1.73 m^2^), and no preliminary evidence of congestion [IVC transversal diameter < 2 cm at US examination (VExUS score of 0), any suspicion of active infection at the first evaluation (body temperature > 37.3°C, and elevated C-reactive protein levels]. Moreover, patients were excluded if diuretic therapy was initiated >12 h after the admission. Demographic data (age, sex, and weight), symptoms (dyspnoea, fatigue, lower limb oedema, and abdominal girth swelling), medical history (comorbidities and medications), vital parameters (heart rate, systolic and diastolic blood pressures, and oxygen saturation), and laboratory tests [haemoglobin, creatinine, sodium, aspartate transaminase (AST), alanine transaminase (ALT), and BNP at admission and before discharge] were collected. Patients underwent multi-organ US assessment according to the VExUS score as previously mentioned within the first 24 h and after 72 h. The first US evaluation was completed in the ED. The 24-h time interval was chosen due to the need for qualified physicians to perform VExUS assessments. The second was completed in the observation unit before either discharge—if the acute condition resolved after a few days—or the admission to a specific hospital department if the patient needed further observation and prolonged intravenous therapy.

The US scanning was performed using an US handheld device (Kosmos EchoNous, Redmond, WA, USA) by two point-of-care US (POCUS)–trained internal medicine fellows and stored in the local archive of the machine to be blindly examined a second time by two physicians (respectively an internal medicine and an emergency medicine physician) board-certified in echocardiography [European Association of Cardiovascular Imaging (EACVI) Certification in Adult Transthoracic Echocardiography] and skilled in POCUS image acquisition and interpretation.

A focus cardiac US (FoCUS) was included at the admission to assess the function of the left and right ventricles.^7^ As the handheld device also provided continuous wave, pulsed wave, and colour flow Doppler capabilities, the evaluation included valvular assessment, according to the international standards defined by the American Society of Echocardiography/EACVI.^8,9^ The device allows users to trace the endocardial borders of the ventricles, obtaining ejection fraction (EF) by biplane Simpson’s method. In addition, all patients underwent transthoracic echocardiography performed with a dedicated machine (Vivid E95 cardiac US) after the ward admission to complete the evaluation of diastolic function and verify the results of the previous scanning.^10^ Since enrolment was performed on a consecutive basis, patient distribution in various VExUS subgroups proved uneven leading to the possibility of dismissing a relevant clinical effect on the basis of statistical insignificance. A thorough perusal of existing literature supports the concept that focusing on patients with maximum VExUS score highlights clinical effects of venous congestion. For example, as demonstrated by Beaubien-Souligny *et al.*^6^ in their study, a VExUS score of 3 is the single predictor with the highest positive likelihood ratio (LR+) and specificity for renal injury.

Based on this premise, we decided to analyse the subgroup of patients with a VExUS score of 3 (severe venous congestion) and compare it with patients having a VExUS score of 2 or lower (mild-to-moderate venous congestion). This allowed a statistically significant and clinically sound comparison between similarly sized groups.

All patients enrolled in the study were followed up with a telephone call at 30 and 60 days after hospital discharge to assess the health status and the rate of hospitalization and death.

### Statistical analysis

Baseline demographic and clinical characteristics were reported as median and interquartile range (IQR) for continuous variables and number and percentage for categorical ones. As explained in the paragraph above, we then pooled the patients to obtain two groups of equal sizes with different congestion statuses and compared group 1 with mild congestion (represented by VExUS 1 and 2 patients) vs. group 2 with severe congestion (represented by VExUS 3 patients). Statistical analysis was carried out using the Mann–Whitney test and Fisher’s exact test. Probability of survival was estimated using the Kaplan–Meier method and compared with the log-rank test. Hazard ratio and its 95% confidence interval were calculated from the Cox regression model. In order to take into account the competitive risks, the proportional hazards model of readmission was estimated using the Fine–Gray model, and the cumulative incidence function was calculated using a Breslow-type estimator. Statistical significance was set at 0.05 probability level for all the tests. All statistical analysis was performed using R version 4.2.1.

## Results

From February 2023 to June 2023, 50 patients fulfilling the above-mentioned inclusion criteria were enrolled, while 8 were excluded (3 had IVC < 2 cm, 3 had Stage V chronic kidney disease, and 2 had fever and elevated C-reactive protein at the admission). Median age was 83 years (IQR 79.25, 88), gender distribution was 58% male vs. 42% female, and predictably the commonest pre-existing condition was cardiovascular illness. The first evaluation was completed within the first 2 h of ED admission. In five cases of delayed VExUS assessment, this was nonetheless performed within the 24-h frame and not >3 h after the first diuretic dose (*Table 1*).

**Table 1:** Demographic and clinical characteristics of the population studied at the admission to the emergency department

Demographic characteristics	
***n***	50
Female/male, *n* (%)	21/29 (42%/58%)
Age, years	83 (79, 88)
Height, cm	169 (158, 173)
Weight, kg	75 (65, 80)
BMI	26 (24, 29)
**Clinical characteristics**	
Hypertension, *n* (%)	37 (74%)
Diabetes, *n* (%)	14 (28%)
Chronic obstructive pulmonary disease, *n* (%)	12 (24%)
Atrial fibrillation *n*, (%)	32 (64%)
Recent myocardial infarction, *n* (%)	1 (2%)
Coronary artery disease, *n* (%)	20 (40%)
**Vital parameters at admission**	
Systolic blood pressure, mmHg	130 (115, 150)
Diastolic blood pressure, mmHg	70 (61, 85)
Heart rate, b.p.m.	88 (71, 105)
Oxygen saturation, %	89 (88, 91)
**First VExUS assessment**	
Admission to VExUS time, h	2 (1, 3)
VExUS before diuretic therapy, %	45 (90%)

Continuous variables are expressed as median (interquartile range) and as percentages for categorical variables.

BMI, body mass index.

### Cardiac point-of-care ultrasound assessment

Median ejection fraction (EF) on admission was 54% (IQR 38.50, 57.00), and only 24% had a moderate-to-severe LV dysfunction. Median pulmonary artery systolic pressure (PAPs) and tricuspid annular plane systolic excursion (TAPSE) were 42 mmHg (IQR 37.00, 52.00) and 15 mm (IQR 14.00, 17.75), respectively. Ninety-eight per cent had tricuspid valve insufficiency, 48% in the moderate range, with a median tricuspid regurgitation velocity of 3 m/s (IQR 2.62, 3.37; *Table 2*).

**Table 2:** Cardiac assessment of the population studied at the admission to the emergency department performed with the handheld device

Cardiac assessment at admission			
EF, %	54 (38, 57)		
EF ≤ 40%, *n* (%)	12 (24%)		
LV mass index, g/m²	104 (91.5, 126.5)		
LA volume index, mL/m²	48.5 (38.75,63.75)		
E/e′	14 (11, 17)		
PAPs, mmHg	42 (37, 52)		
TRV, m/s	3 (2.62, 3.37)		
TAPSE, mm	15 (14, 17)		
**Valve disease**			
MVI, *n* (%)	Mild 28 (56%) Moderate 10 (20%) Severe 4 (8%)	MVS, *n* (%)	Mild 1 (2%) Moderate 2 (4%) Severe 0
AVI, *n* (%)	Mild 25 (50%) Moderate 2 (4%) Severe 2 (4%)	AVS, *n* (%)	Mild 2 (4%) Moderate 2 (4%) Severe 3 (6%)
TVI, *n* (%)	Mild 11 (22%) Moderate 24 (48%) Severe 14 (28%)	TVS, *n* (%)	Mild 0 Moderate 0 Severe 0
PVI, *n* (%)	Mild 21 (42%) Moderate 0 Severe 0	PVS, *n* (%)	Mild 0 Moderate 0 Severe 0

In addition, all patients underwent transthoracic echocardiography after the first admission to complete the evaluation of diastolic function and verify the results. Continuous variables are expressed as median ± interquartile range and percentages for categorical variables.

EF, ejection fraction; LV, left ventricle; LA, left atrial; E, trans-mitral E-wave; e′, early diastolic velocity; PAPs, systolic pulmonary artery pressure; TRV, tricuspid regurgitation velocity; TAPSE, tricuspid annular plane systolic excursion; MVI, mitral valve insufficiency; MVS, mitral valve stenosis; AVI, aortic valve insufficiency; AVS, aortic valve stenosis; TVI, tricuspid valve insufficiency; TVS, tricuspid valve stenosis; PVI, pulmonary valve insufficiency; PVS, pulmonary valve stenosis.

### Venous excess ultrasound score and Doppler assessment

*Table 3* shows the results of VExUS assessment at admission to the ED and 72 h after. On admission, more than half of the patients had severe venous congestion (VExUS score of 3). After 72 h, after diuretic therapy was initiated, 32% had no more signs of congestion at the Doppler VExUS examination (IVC < 2 cm, VExUS score of 0); a similar percentage still exhibited a severe grade of congestion despite therapy.

**Table 3:** Venous excess ultrasound assessment of the population studied at the admission to the emergency department and at the reassessment (72 h after admission)

VExUS score at admission and at reassessment		
Variable	Admission Day 1	Reassessment Day3
IVC transverse diameter, cm	2.40 (2.20, 2.62)	2.18 (1.80, 2.35)
HV Doppler		
Normal, *n* (%)	9 (18%)	21 (42%)
Mildly abnormal, *n* (%)	3 (6%)	3 (6%)
Severely abnormal, *n* (%)	38 (76%)	26 (52%)
PV Doppler		
Normal, *n* (%)	5 (10%)	21 (42%)
Mildly abnormal, *n* (%)	21 (42%)	16 (32%)
Severely abnormal, *n* (%)	24 (48%)	13 (26%)
Portal vein pulsatility index	0.49 [0.42, 0.67]	0.40 [0.12, 0.50]
Renal Doppler		
Undetectable, *n* (%)	3 (6%)	3 (6%)
Normal, *n* (%)	6 (12%)	19 (38%)
Mildly abnormal, *n* (%)	23 (46%)	16 (32%)
Severely abnormal, *n* (%)	18 (36%)	12 (24%)
VExUS score		
No congestion, *n* (%)	0 (0%)	16 (32%)
VExUS 1, *n* (%)	11 (22%)	7 (14%)
VExUS 2, *n* (%)	11 (22%)	12 (24%)
VExUS 3, *n* (%)	28 (56%)	15 (30%)

Continuous variables are expressed as median ± interquartile range and percentages for categorical variables.

IVC, inferior vena cava; HV, hepatic vein; PV, portal vein.

### Laboratory tests

The median creatinine, eGFR, and BNP levels at admission were 1.22 mg/dL (IQR 0.93, 1.97), 49.50 mL/min/1.73 m^2^ (IQR 30.00, 64.50), and 994.50 pg/mL (IQR 641.00, 1625.50), respectively, as shown in *Table 4*. All the laboratory test variations were essentially homogeneous with clinical and US reassessments at 72 h.

**Table 4:** Laboratory test at admission and reassessment (72 h after emergency department admission)

Laboratory test at admission and at reassessment		
Variable	Day 1 admission	Day 3 reassessment
Hb, g/dL	11.90 (10.72, 12.60)	11.90 (10.62, 12.85)
AST, UI/L	34 (25, 48)	27 (20, 35)
ALT, UI/L	22 (12, 32)	18 (11, 28)
Sodium, mmol/L	139 (135, 141)	140 (137, 142)
Creatinine, mg/dL	1.22 (0.93, 1.97)	1.23 (1.04, 1.76)
eGFR, mL/min	49 (30, 64)	48 (30, 62)
BNP, pg/mL	994 (641, 1625)	NA

Continuous variables are expressed as median ± interquartile range and percentage for categorical variables. BNP was measured only at admission as an inclusion criteria.

Hb, haemoglobin; AST, aspartate aminotransferase; ALT, alanine aminotransferase; eGFR, estimated glomerular filtration rate; BNP, brain natriuretic peptide.

### Hospitalization and death after admission to the emergency department and during follow-up

Eighty per cent of patients were hospitalized after admission to the ED, with the majority in the internal medicine unit (42%). Median hospital stay was 10 days. Of these, 85% (34 patients) were discharged while 6 died in-hospital. All of these six exhibited a first-assessment VExUS score of 3 in the ED. No patient with a VExUS score < 3 died during the study. During short-term follow-up, 18 patients were readmitted to the ED and hospitalized with a diagnosis of acute decompensated HF. Median time to readmission was 34 days, and 94% of the readmitted patients had a VExUS score of 3 at the Doppler assessment at the first ED admission (*Table 5*). Readmission rates and cumulative readmission incidence rates for the population studied were consistent with the data from the most recent epidemiological studies.^11^ When readmission rates were adjusted for VExUS score, VExUS 3 population had a statistically significant high probability of readmission (*P* < 0.001; Supplementary material online, *Table S1*).

**Table 5:** Patients hospitalized and discharged after the first assessment in the emergency department and patients readmitted and hospitalized during follow-up

Variable	*n*
Hospitalization after first admission to ED, *n* (%)	40 (80%)
Day of hospitalization, median (IQR)	10 (8, 13)
Discharge after hospitalization, *n* (%)	34 (85%)
Death	6 (15%)
VExUS 3	6 (100%)
VExUS 2	0
VExUS 1	0
Readmission to ED during the follow-up^a^, *n* (%)	18 (36%)
VExUS 3^b^	17 (94.4%)
VExUS 2^b^	1 (5.6%)
VExUS 1^b^	0
Days to readmission from first access to ED, median (IQR)	34 (25, 46)
Death, *n* (%)	2 (11%)
VExUS 3	2 (100%)
VExUS 2	0
VExUS 1	0

^a^Readmission rate for the total observation time, 200 days.

^b^VExUS grade at the first VExUS evaluation of the patients readmitted to the ED during the follow-up.

### Comparison between venous excess ultrasound 1–2 patients and venous excess ultrasound 3 patients

**Figure 1:**
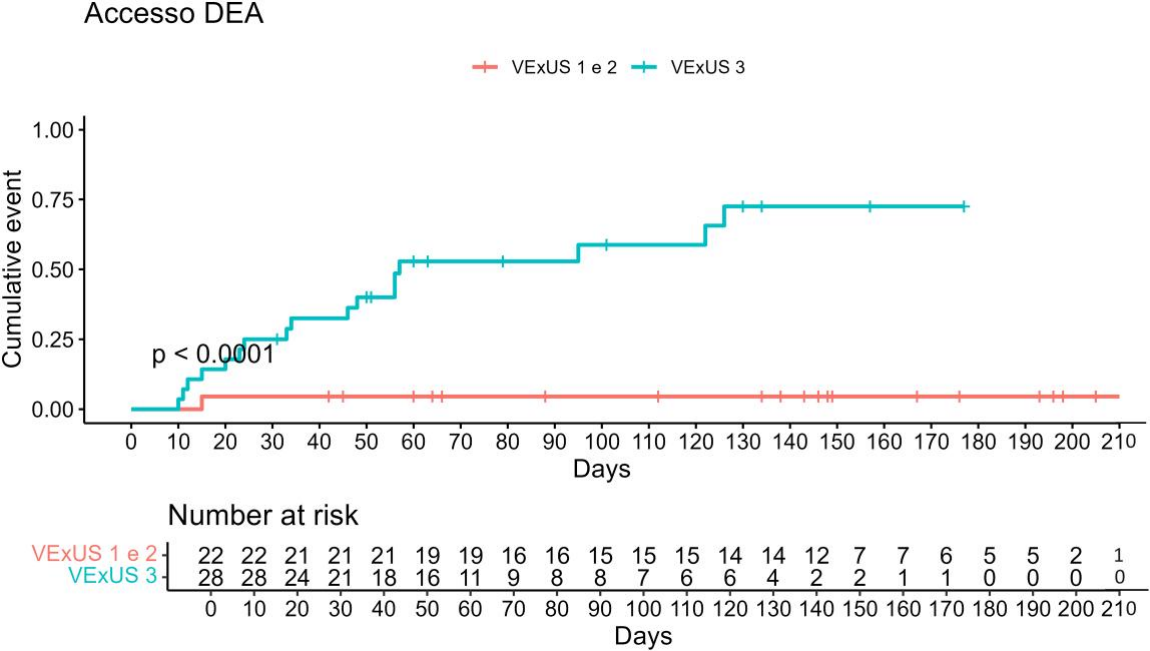
The Kaplan–Meier curve for the probability of readmission comparing mild-to-moderate congestion (venous excess ultrasound score of 1–2) and severe venous congestion (venous excess ultrasound score of 3).

**Figure 2:**
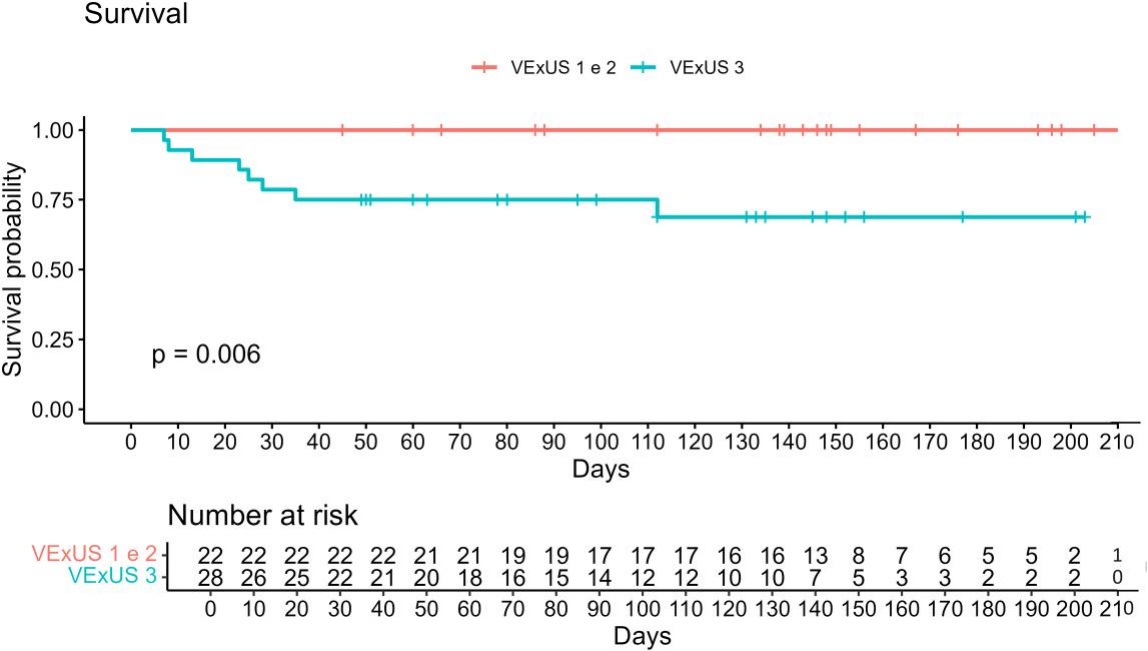
The Kaplan–Meier curve for the probability of survival comparing mild-to-moderate congestion (venous excess ultrasound score of 1–2) and severe venous congestion (venous excess ultrasound score of 3).

**Figure 3:**
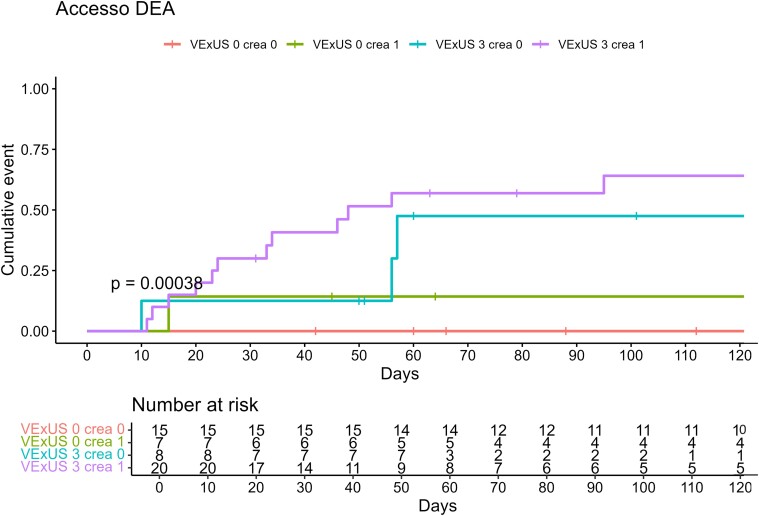
The Kaplan–Meier curve for the probability of readmission comparing mild-to-moderate congestion (venous excess ultrasound score of 1–2) and severe venous congestion (venous excess ultrasound score of 3) adjusted for creatinine values. VExUS 1–2 norm, patients with venous excess ultrasound score of 1–2 with normal creatinine values (≤1.2 mg/dL); VExUS 1–2 alt, patients with venous excess ultrasound score of 1–2 with altered creatinine values (>1.2 mg/dL); VExUS 3 norm, patients with venous excess ultrasound score of 3 with normal creatinine values (≤1.2 mg/dL); VExUS 3 alt, patients with venous excess ultrasound score of 3 with altered creatinine values (>1.2 mg/dL).

Patients were enrolled on a consecutive basis, and, as a consequence, distribution among VExUS score groups proved uneven. In order to investigate re-hospitalization and death due to HF, both indicators of severe disease, we chose to cluster patients with mild and moderate venous congestion, thus forming and comparing two groups with similar sample size, as previously reported in the *Statistical analysis* section (VExUS 1–2 vs. VExUS 3, 22 patients vs. 28 patients). The Kaplan–Meier curves in *Figures 1* and *2* highlight, respectively, readmission probability and survival at the 200-day time interval, showing in both cases statistically significant differences between the two groups. One hundred days after the first ED admission, the probability of readmission was 55% for VExUS 3 patients and 5% for the VExUS 1–2 group (*P* < 0.0001). Furthermore, the survival curve shows a 100% survival rate for VExUS 1–2 patients vs. 75% for patients with severe venous congestion 100 days after the first US examination (*P* = 0.006). When readmission rates were adjusted for creatinine values, VExUS 3 population with altered creatinine (creatinine > 1.2 mg/dL) had a statistically significant higher probability of readmission compared to VExUS 1–2 population with elevated creatinine values (*P* < 0.001, *Figure 3*). As for secondary endpoints, glomerular filtrate and creatinine values were statistically different between the two groups, whereas there were no statistically significant differences in BNP admission values (855 vs. 1155 pg/mL, *P*-value of 0.12, *Table 6*).

**Table 6:** Comparison of biochemical and ultrasound parameters and hospital readmission and death for heart failure between patients with venous excess ultrasound score of 1–2 and patients with venous excess ultrasound score of 3

Variable	VExUS 1–2	VExUS 3	*P*-value
***n***	22	28	
Death (*n*, %)	0	8 (28.6%)	0.006
Readmission (*n*, %)	1 (4.5%)	16 (57.1%)	<0.001
eGFR, mL/min	54 (45, 68)	31 (27, 59)	0.026
Creatinine, mg/dL	1.06 (0.90, 1.20)	1.54 (1.15, 2.12)	0.008
BNP, pg/mL	855 (552, 1487)	1155 (707, 1760)	0.116
HV Doppler (*n*, %)			
Normal	9 (40.9%)	0	<0.001
Mildly abnormal	3 (13.6%)	0	
Severely abnormal	10 (45.5%)	28 (100%)	
PV Doppler (*n*, %)			
Normal	4 (18.2%)	1 (3.6%)	<0.001
Mildly abnormal	17 (77.3%)	4 (14.3%)	
Severely abnormal	1 (4.5%)	23 (82.1%)	
Renal Doppler (*n*, %)			
Undetectable	0	3 (10.7%)	<0.001
Normal	5 (22.7%)	1 (3.6%)	
Mildly abnormal	17 (77.3%)	6 (21.4%)	
Severely Abnormal	0	18 (64.3%)	

Continuous variables are expressed as median ± interquartile range and percentage for categorical variables.

eGFR, estimated glomerular filtration rate; BNP, brain natriuretic peptide; HV, hepatic vein; PV, portal vein.

### Comparison of biochemical and ultrasound parameters in patients with no readmission or death vs. readmission or death

In our population, patients readmitted to the ED during the follow-up or deceased after the first evaluation were 24, i.e. 48% of the sample studied. In this relevant subset, age, most biochemical and cardiac POCUS parameters were substantially similar to the non-readmission/death group, while eGFR, creatinine, and TAPSE showed a statistically significant difference (*Table 7*). In particular, renal function was lower in patients readmitted or deceased after the first assessment. Comparing the Doppler evaluation, ∼96% of readmitted or dead patients had VExUS score of 3 at admission, with severely abnormal portal and renal Doppler in most cases.

**Table 7:** Comparison of biochemical and ultrasound parameters in patients with no readmission or death vs. readmission or death

Variable	No readmission or death	Readmission or death	*P*-value
*n*	26	24	
Age	82.50 (76.75, 88.75)	83.50 (80.00, 87.00)	0.846
Laboratory test			
Hb, g/dL	12.1 (10.8, 13.4)	11.8 (10.6, 12.4)	0.398
AST, UI/L	36 (26, 50)	30 (21, 42)	0.294
ALT, UI/L	24 (16, 38)	14 (8, 28)	0.082
Creatinine, mg/dL	1.11 (0.90, 1.35)	1.72 (1.23, 2.12)	0.006
eGFR, mL/min	55 (37, 68)	31 (27, 54)	0.024
BNP, pg/mL	884 (574, 1622)	1075 (704, 1607)	0.461
Cardiac POCUS assessment			
EF, %	55 (40, 59)	46 (34, 55)	0.078
TAPSE, mm	15 (15,18)	14 (12, 16)	0.025
PAPs, mmHg	42 (40, 51)	44.00 (36, 57)	0.719
Doppler assessment and VExUS score			
HV Doppler (*n*, %)			
Normal	9 (34.6%)	0	<0.001
Mildly abnormal	3 (11.5%)	0	
Severely abnormal	14 (53.8%)	24 (100%)	
PV Doppler (*n*, %)			
Normal	4 (15.4%)	1 (4.2%)	<0.001
Mildly abnormal	17 (65.4%)	4 (16.7%)	
Severely abnormal	5 (19.2%)	19 (79.2%)	
Renal Doppler (*n*, %)			
Undetectable	0	3 (12.5%)	<0.001
Normal	5 (19.2%)	1 (4.2%)	
Mildly abnormal	17 (65.4%)	6 (25%)	
Severely abnormal	4 (15.4%)	14 (58.3%)	
VExUS score (*n*,%)			
VExUS score 1	11 (42.3%)	0	<0.001
VExUS score 2	10 (38.5%)	1 (4.2%)	
VExUS score 3	5 (19.2%)	23 (95.8%)	

Continuous variables are expressed as median ± interquartile range and percentage for categorical variables.

Hb, haemoglobin; AST, aspartate aminotransferase; ALT, alanine aminotransferase; eGFR estimated glomerular filtration rate; BNP, brain natriuretic peptide; EF, ejection fraction; TAPSE, tricuspid annular plane systolic excursion; PAPs, systolic pulmonary artery pressure; E, trans-mitral E-wave; e′, early diastolic velocity; HV, hepatic vein; PV, portal vein.

## Discussion

This is the first observational study analysing a cohort of patients with acute decompensated HF in the ED using a handheld US system to evaluate the feasibility and the prognostic value of the VExUS score.

In our study, readmission and death due to HF in patients with acute decompensated HF were associated with (i) severe venous congestion, (ii) impaired renal function, and (iii) poor right ventricle (RV) systolic function. Different survival rates at follow-up were not due to age difference between the subsets, as average age was almost identical to the two groups. Similarly, laboratory test values such as haemoglobin, transaminases, and BNP showed no significant differences between the two groups (*Table 7*). In our study, the only statistically different parameters between patients with and without events were renal function, TAPSE, and the VExUS score. Most interestingly, in our population, the probability of both readmission and death was significantly higher in patients with a VExUS score of 3 than those with lower values.

About 96% of patients readmitted to the ED or deceased during follow-up scored VExUS 3 at the first Doppler evaluation, presenting a decreased renal function (eGFR 54 vs. 31 mL/min/1.73 m^2^, *P*-value of 0.024) and lower TAPSE (15.5 vs. 14.5 mm, *P*-value of 0.025) compared with the other subgroup. As known, chronic kidney disease and worsening of renal function are linked to poor outcomes in HF.^12^ In our study, we found a worsening renal function in the event population that mostly exhibited with severe congestion present on admission at the ED. We took into account that higher mortality was due to decreased renal function and consequently assessed if all mortality excess was due to renal impairment. As glaringly shown in *Figure 2*, it is evident that congestion constitutes, in itself, an additive cause of mortality.

This supports the predominant role of venous congestion rather than the reduction of cardiac output in the development of worsening renal function in patients with decompensated HF, as the first elevation of central venous pressure can be transmitted backwards to the renal veins, resulting in direct impairment of renal function. We chose to analyse the subgroup of patients with a VExUS score of 3 (severe venous congestion) and compare it with patients with a VExUS score of 2 or lower (mild-to-moderate venous congestion) as a thorough review of the current literature supports that focusing on patients with the highest VExUS score highlights the clinical implications of venous congestion.^6^

When comparing the impaired renal function VExUS 3 population with VExUS 1–2 population with similarly impaired renal function, readmission rate is still statistically higher in the severe venous congestion group (*Figure 3*). Accordingly, a previous large cohort study on AHF patients found that patients who developed worsening renal function had a greater central venous pressure (CVP) either on admission or after full medical therapy.^13^

This has relevant clinical implications. Since congestion is an additive, albeit probably not independent, mortality factor, it is likely that maximal patient benefit comes from pushing decongestion, targeting at least a 1-point VExUS decrease before hospital discharge. This constitutes a novel strategy in the management of HF patients. Our study supports that RV dysfunction contributed to the harmful effects of volume overload (expressed by a higher VExUS score) and is associated with more impaired renal function and a higher risk of hospital readmission and death due to HF. Advanced venous congestion and reduced systolic RV function are the combined factors determining whether renal function deteriorates in acute decompensated HF.^14^

In support of this, only one patient in the event subgroup had a VExUS score < 3, and 5% of VExUS 1–2 group patients were readmitted to ED for acute decompensated HF 100 days after the first admission. These results underline that the VExUS score can predict the risk of readmission and death in patients with acute decompensated HF, identifying patients with right HF that could lead to multi-organ dysfunction and death.

Before our study, only Torres-Arrese *et al.*^15,16^ assessed the role of the VExUS score in AHF patients’ prognosis. In accordance with our findings, they found that a VExUS score of 3 at first evaluation can predict mortality during admission, HF-related death, and early readmission. On the other hand, Torres-Arrese *et al.* found that an IVC > 2 cm (area under the curve (AUC) 0.758, sensitivity 93 %, and specificity 58.3%) and the presence of an intra-renal monophasic pattern (AUC 0. 834, sensitivity 91.7%, and specificity 67.4%) in the follow-up visits predicted AHF-related readmission.

As known, the intra-renal venous Doppler is technically difficult to obtain, leading to suboptimal recordings. It is also possible that parenchymal renal disease could alter the intra-renal Doppler venous waveforms.^17^ Based on our data, the use of IVC diameter > 2 cm and of an intra-renal monophasic pattern as the parameters predicting poor outcome can be misleading as the presence of these two variables would lead to a VExUS score of only 2, which, in our population, did not correlate with readmission or death during follow-up (*Figures 2* and *3*).

In our population, a severely abnormal renal Doppler pattern demonstrates high specificity for severe congestion and poor outcomes, as no patients in the VExUS 1–2 group showed this alteration (*Table 6*). However, despite its specificity, relying solely on renal Doppler for congestion assessment may be insufficient to accurately define the congestive status, and it may not always be interpretable. This is evident when comparing Doppler patterns between patients who died (either during hospitalization or follow-up) or experienced readmission to the ED and those who survived or had no additional ED admission. In the poor outcome cohort, severe congestion patterns are most prevalent across all examined anatomical districts (*Table 7*). Therefore, a comprehensive examination is recommended to accurately determine the degree of venous congestion.

As for feasibility, in our study, Doppler evaluation was correctly performed and adequately interpreted in most cases. In three patients, it was not possible to acquire adequate renal Doppler images. Despite this lack, they were nonetheless included in the study since both the hepatic vein and portal vein Doppler showed a severely altered pattern both on admission and after 72 h, thus classified as VExUS 3 independently of the renal venous pattern. The Doppler examination could be completed swiftly in most cases, even in the emergency setting providing crucial clinical information for the patient’s management. As obtaining a clear renal Doppler tracing is more challenging, being highly influenced by acoustic window quality and severe congestion can also be determined by hepatic and portal flow alone, it seems reasonable to defer the evaluation of a simplified score not involving renal Doppler to future studies. We believe that the renal Doppler alteration, which is associated with longer-lasting and more severe congestion, has at present to be maintained in the evaluation.

It is essential to acknowledge that our study has certain limitations. First, this is an observational research study with a limited number of patients enrolled. Second, considering the patient’s age, the follow-up did not include a US evaluation but only a telephone follow-up to record the HF-related events. The study has been planned and performed to assess the validity of the VExUS score as a predictor of severity of HF and relapse risk. As such, it is not aimed at assessing therapeutic decongestion efficacy of a certain drug or procedure, so use of diuretic therapy, vasopressor, or inotropes during the acute phase was not further analysed for our purpose. The crucial focus is to evaluate the feasibility of a VExUS-driven approach in the management of AHF patients. We plan to prove or disprove these results with a larger multicentre study also comparing a VExUS-driven vs. non–VExUS-driven clinical strategy to provide conclusive information on the therapeutic implications of the VExUS score in these HF patients.

## Conclusions

This study is to our knowledge the first to provide VExUS score validation in an ED population. We deliberately focused on patients admitted for suspected HF, in order to have a homogeneous study population, but in all likelihood, VExUS can be reliably performed on any patient presenting to the ED with a clinical suspicion of venous congestion. This includes patients with renal insufficiency, iatrogenic fluid overload, etc.

As such, our study showed that severe venous congestion, defined as a VExUS score of 3 at the initial assessment of patients admitted to ED with acute decompensated HF, predicts inpatient mortality, HF-related death, and early readmission. Secondly, we demonstrated that severe venous congestion, lower RV systolic function, and worsened renal function are associated with poor outcome (be it readmission or death due to HF). Finally, in our study, the VExUS score was found to be technically feasible in almost all patients, despite tachypnoea and incomplete cooperation, proving feasibility and adequate interpretation also in an ED setting.

The use of bedside US clinical evaluation is critical for identifying, monitoring, and assessing the response to treatment of venous congestion. In addition to echocardiography and lung US, VExUS multi-organ venous Doppler assessment could provide further insight on which patients are likely to tolerate and benefit from fluid removal and to establish the extent of diuresis or other decongesting therapeutic options thus optimizing the outcome in acute decompensated HF patients.

## Lead author biography


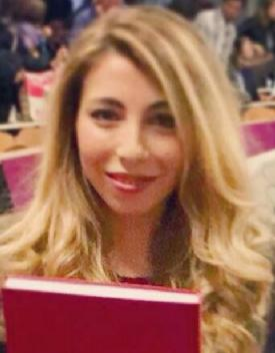


Dr Irene Landi is an internal medicine specialist currently working in the emergency department. She compounds her emergency medical skills with a solid knowledge in elective echocardiography. She has authored research articles focusing on mitral valve prolapse and heart failure. She now blends these two fields of interest researching the role of point-of-care ultrasound (PoCUS) in the management of acute heart failure patients.

## Supplementary Material

oeae050_Supplementary_Data

## Data Availability

The data that support the findings of this study are available from the corresponding author, M.G., upon reasonable request.
