# Supplementary material for: Assessment of venous congestion with venous excess ultrasound score in the prognosis of acute heart failure in the emergency department: a prospective study
Source: Eur Heart J Open. 2024 Jul 10;4(5):oeae050. doi: 10.1093/ehjopen/oeae050 (PMC11373564; doi:10.1093/ehjopen/oeae050)
Supplement: oeae050_Supplementary_Data [file oeae050_supplementary_data.docx]

**Supplementary Materials**

| **Variable** | **N** | |
| --- | --- | --- |
| **Re-admission to ED during the total observation time * n, (%)**  **Re-admission rate (95% CI) per 10.000 patients-days ***  **Re-admission to ED at 90 days of follow-up n, (%)**  **Re-admission rate (95% CI) per 10.000 patients-days at 90 days of follow-up** | 18, (36)  38,6 (CI 22,9-70)  15, (30)  29 (CI 17-43) | |
| **VExUS 1-2 re-admission rate (95% CI) per 10.000 patients-days at 90 days of follow-up** | **VExUS 3 re-admission rate (95% CI) per 10.000 patients-days at 90 days of follow-up** | **p-value** |
| 5 (CI 0-14) | 60 (CI 43-79) | < 0.001 |

**Table 1.** Re-admission rates and cumulative re-admission incidence rate for the population studied adjusted for VExUS score. * total observation time, 200 days.
